# Evaluation of Morphological and Osteometric Sex Estimation on Historical Skeletal Material of Subadult Individuals From Germany

**DOI:** 10.1002/ajpa.70308

**Published:** 2026-07-08

**Authors:** Clara L. Behrendt, Katharina M. J. Tenorio, Susanna Hixt, Janine Mazanec, Susanne Hummel, Birgit Grosskopf

**Affiliations:** ^1^ Historical Anthropology and Human Ecology, Institute of Zoology and Anthropology University of Göttingen Göttingen Germany; ^2^ Institute of Legal Medicine Hannover Medical School Hannover Germany; ^3^ Institute of Infectious Diseases and Infection Control University Hospital Jena Jena Germany

**Keywords:** ilium, mandible, osteometry, sex estimation, subadults

## Abstract

**Objectives:**

While sex estimation based on morphological features of the adult skeleton is considered reliable, it proves to be challenging, especially in skeletons of subadults. The purpose of this study is to investigate the accuracy of sex estimation of the methods described below and errors within the observer and between observers.

**Materials and Methods:**

For this purpose, samples from 20 individuals from four different skeleton series from Lower Saxony (Germany) were analyzed. DNA‐analytical examinations were used for sex determination. The primary aim was to validate the morphological and osteometric method applied to the Facies auricularis as described by Luna et al. (2017, 2021), alongside the discriminant function analyses for the Os ilium and mandible outlined by Schutkowski (1990). The morphological and osteometric procedures were performed as a blind study and as an inter‐observer study.

**Results:**

Contrary to the results from previous studies, none of the methodologies proved to be suitable for reliable sex estimation on the skeleton of subadults. However, the discriminant functions (DF) according to Schutkowski (1990) show a higher certainty of determination than the methodology proposed by Luna et al. (2017, 2021).

**Discussion:**

Methods for sex estimation in subadult individuals hold considerable promise. However, their validity and transferability to other populations and time periods remain problematic. Demographic factors and population‐specific differences result in varying levels of accuracy.

## 1 Introduction

A complete individual profile which includes the sex is relevant in forensic bioarchaeology and paleodemographic investigations, regardless of individual age. One significant domain in which such paleodemographic analyses find application is the examination of humanitarian crises and historical events. For instance, the discovery of mass burial sites of children was reported associated with Canadian Residential Schools, where cultural genocide of aboriginal children took place until 1996 (Potvin 2015; Austen 2021), with workhouses during the Great Famine in Ireland (Geber 2016) and with Mother and Child homes in Ireland (Corless 2012; Buckley and McGregor 2019). War profoundly elevates infant mortality rates as well through direct violence, destruction of healthcare systems, malnutrition, and disease (Horiuchi 1983; Savitz et al. 1993; Wagner et al. 2018). For example, 20% of the deaths during the Guatemalan Armed Conflict from 1960 to 1998 were children (Lewis and Flavel 2006).

An essential part of such investigations is the determination of the sex. This is often done as a first step since some methods for reconstruction of stature and age at death are based on the knowledge of the sex. In skeletons of adults, sex estimation can be easily performed due to distinct sexually dimorphic features induced by the sex hormones. They are manifested especially in the pelvis, long bones, and skull where an accuracy of sex estimation of 90% and above is common (Herrmann et al. 1990). In skeletons of subadults, sex estimation is more difficult, as the sex dimorphism is less pronounced and develops with the onset of puberty.

For sex estimation of skeletons of subadults, methods based on morphology, morphometry, and osteometry are used. Hormonal levels control the development of genital organs during fetal and neonatal life and thus influence skeletal dimorphism (Lanciotti et al. 2018). Therefore, most studies were focused on skeleton sites where dimorphism is especially evident in adults. In the pelvis, the accuracy of the arch criteria was 82.3%, the sciatic notch 80.7%, the depth of the sciatic notch 79%, and the mandibular arcade shape 77.6% (Sutter 2003), which are above the minimum standard accuracy of 75% recommended by De Vito and Saunders (1990).

The discriminant analytical method for sex estimation of fetal and neonatal individuals developed by Schutkowski (1990) involves the measurement of various skeletal dimensions, including the widths and depth of the sciatic notch in the Os ilium. A similar approach is applied to the mandible, utilizing measurements of its width, length, and height for discriminant analyses. This method was validated using known sexes of 43 female and 61 male fetuses and neonates, achieving a success rate of more than 70%. Sutter (2003) and Vlak et al. (2008) obtained less promising results when applying this method to pre‐Columbian mummies and a historic Portuguese skeletal series, respectively, demonstrating that the applicability of Schutkowski's (1990) method may vary across different populations and contexts.

In 2017, a new method was developed, combining morphological, osteometric, and discriminant analytical approaches on the Os ilium (Luna et al. 2017). This method focuses on the surface structure of the Facies auricularis and captures various qualitative and quantitative features. By analyzing measurement paths, angles, their ratios, and integrating these values into a discriminant function, this method allowed a high degree of accuracy in sex estimation of subadult individuals.

Prior research has demonstrated the efficacy of the auricular surface method, as developed by Luna et al. (2017), in estimating the sex of subadult skeletons. The method has been applied by Monge Calleja et al. (2020) and Marino et al. (2021) on different skeletal series, with varying levels of success being reported in each case. Monge Calleja et al. (2020) observed that the method demonstrated particular efficacy for male individuals under the age of two within the Lisbon Collection, attaining accuracy rates exceeding 85%. Similarly promising results were demonstrated by Marino et al. (2021) for children aged 4–12 in their Italian skeletal series, which highlighted the effectiveness of the method in detecting sexual dimorphism as it becomes more pronounced with age.

Nevertheless, the findings of the two studies were contingent on the demographic characteristics of the respective skeletal collections. While these skeletal collections originated from the same period, their applicability to historic skeletal series, such as those from medieval contexts, is uncertain. Consequently, the validation of such samples is of particular importance to assess the method's transferability and reliability across different populations and time periods as differences in environmental conditions, socio‐cultural factors, and temporal distance may influence the expression of dimorphic traits (Hall 1978; Johnston and Zimmer 1989; Scheuer and MacLaughlin‐Black 1994; Belcastro et al. 2019).

This investigation aimed to evaluate the accuracy of the morphologic and osteometric method as described by Luna in subadult skeletons from Medieval to Early Modern Times in Lower Saxony. For comparison, the morphometric method by Schutkowski (1990) was included. As an unequivocal reference method, Short tandem repeats (STR) analyses were performed to determine the genotype.

## 2 Material

The material analyzed in this study comprises skeletal remains of 20 subadults, which are held at the Institute of Historical Anthropology and Human Ecology (University of Göttingen). These samples originate from various historical periods and locations in Lower Saxony, Germany. Institutional permission to access and study the remains was granted by the Historical Anthropology Collection at the University of Göttingen. For details regarding ethical considerations, see the Ethics Statement. The skeletal series are briefly described below, with the sample composition presented in Table 1. Subadult individuals were included if their estimated age at death fell within the anthropological age classes neonate, Infans I, or Infans II. Inclusion further required adequate preservation of the Os ilium and mandible, specifically an intact and clearly demarcated Facies auricularis for the application of the method by Luna et al. (2017, 2021), and surface preservation free of erosion for the methods described by Schutkowski (1990).

**TABLE 1:** Distribution of age at death of the individuals.

Skeletal series	Age at death		
	0–24 months	2–6 years	7–12 years
Eldagsen (EL)	1 (EL 10)	2 (EL 117, EL 137)	2 (EL 14, EL 218)
Göttingen Campus (Gö C)	—	1 (Gö C 38)	1 (Gö C 105a)
Medenheim (MH)	4 (MH 3, MH 14, MH 38, MH 41)	2 (MH 12, MH 69)	1 (MH 50)
St. Nikolaikirche (N)		5 (N 1, N 8, N 11, N 12, N 15)	1 (N 6)
Total	5	10	5

### 2.1 Eldagsen (EL)

In 2013, human skeletal remains were discovered near St. Alexandri Church in Eldagsen, Lower Saxony (Germany). A total of 356 burials were recorded, with the majority dating to the early modern period (16th–18th century). Human skeletal remains were recovered during preventive archaeological excavations carried out by ArchaeoFirm Poremba & Kunze GbR under the direction of Stefan Agostinetto, prompted by the redesign of the town's market square. The excavation covered a total area over 1500 m^2^ and documented 413 features, comprising 57 buildings and settlement structures alongside 356 burials, located primarily in the north and northeast of the church. According to excavation records, the cemetery was in active use from the 9th to the 19th century. Individual graves varied in shape and size but were consistently shallow and densely spaced, reflecting the long‐term and intensive use of the burial ground over several centuries. The demographic composition of the series spans both sexes across all age classes (Frischalowski et al. 2015). From this skeletal collection, five subadult individuals meeting the inclusion criteria outlined above were selected in the present study.

### 2.2 Göttingen Campus (Gö C)

The skeletal series referred to as Göttingen Campus comprises remains recovered from a former catholic cemetery that served as burial ground of the catholic parish of St. Michaelis, Göttingen (Germany), between 1851 and 1889 (Modern Era). The cemetery primarily served the catholic minority community within a predominantly Protestant urban environment. The individuals interred were largely drawn from the lower strata of urban society, making this skeletal collection representative of the poorer urban population of 19th century Germany. Following the discovery of the site during construction works, a portion of the cemetery was archaeologically excavated and scientifically investigated. A total of 151 individuals were subjected to anthropological analysis, comprising 114 adults, 10 juveniles, and 27 additional subadult individuals. The sex distribution reveals a markedly elevated masculinity index, which is attributed to the selective transfer of destitute male patients to the university's anatomical institute, as evidenced by dissection traces observed on 35 individuals. The overall preservation of the skeletal remains is predominantly good. However, brushite formation and skeletal degradation were observed mainly on the skeletal remains of subadult individuals (Grosskopf 2015). From this skeletal collection, two subadult individuals were selected in the present study.

### 2.3 Medenheim (MH)

The Medenheim skeletal series derives from an archaeological rescue excavation conducted in the 1990s prior to construction work, with the direct involvement of researchers from the Department of Historical Anthropology at the University of Göttingen. Medenheim was a medieval deserted settlement of rural character, located at the southern edge of the present‐day town of Northeim, Lower Saxony (Germany). The parish cemetery of St. Bonifatius church was in use from the late 11th or early 12th century until the reconsecration of the church in 1388. A total of 137 skeletal individuals and 15 disarticulated bone finds were recovered, of which 79 individuals were subjected to anthropological analysis. The overall preservation of the skeletal remains is good, and the majority of individuals exhibit no or only minor signs of decomposition. The demographic composition of the series spans both sexes across all age classes. More than half of the individuals died in subadulthood, with Infans I most strongly represented, a pattern consistent with the mortality profile expected for historical populations. The pathological spectrum reflects the harsh living conditions of a medieval rural community and includes evidence of scurvy, tuberculosis‐related lesions, and degenerative and arthritic skeletal changes (Stavenhagen et al. 2021). From this skeletal series, seven individuals meeting the inclusion criteria were selected for the present study.

### 2.4 St. Nikolaikirche (*N*)

Archaeological excavations at the former site of the medieval church of St. Nikolai in Göttingen, Lower Saxony (Germany), revealed human skeletal remains. The burials were located both within the church interior and in the adjacent exterior areas to the north and south. All documented burials were oriented in the standard west–east position and showed no discernible differences in funerary rite. While precise burial dates remain indeterminable, they are estimated to span from the medieval to the modern period. The preservation of the skeletal remains is variable, ranging from complete skeletons to heavily fragmented material. Initial anthropological analyses indicated that the collection encompasses individuals of male and female sex across all anthropological age classes (Arndt 2012). From this skeletal collection, six individuals meeting the inclusion criteria were selected for the present study.

## 3 Methods

The skeletal elements to be analyzed were independently measured or morphologically evaluated for each methodology by three researchers. The methods following Luna et al. (2021) were carried out in a reduced manner as the selected traits and osteometric measures showed the most promising results.

The intra‐ and interobserver comparison serves to verify the reproducibility of the methodology. To assess intra‐ and interobserver reliability, Cohen's kappa was utilized as in the original studies. Interpretation of Cohen's kappa was followed by Landis and Koch (1977). The reliable sex determination is based on the gonosomal‐specific STR analysis, which allows a positive determination for both female and male individuals (Nováček and Grosskopf 2021).

### 3.1 Morphological Assessment (Luna et al. 2021)

The morphological evaluation of the Facies auricularis as described by Luna et al. (2021) was conducted in a blinded manner and each researcher evaluated the Overall Morphology (OM) three times. To ensure a blinded design, researchers were provided with no prior information regarding age, sex, or skeletal series origin of the Ossa ilia. Each Facies auricularis was independently assessed on three separate occasions, with evaluations spaced at least two days apart. According to the authors, a right angle indicates female sex, while an obtuse angle suggests male sex.

### 3.2 Osteometric Method (Luna et al. 2021)

Standardized photographs of the left and right Facies auricularis were taken at a fixed distance (a = 20 cm) for the objective osteometric method per Luna et al. (2021). Images were aligned in an image editor such that the anterior and inferior margins of the Facies auricularis were positioned horizontally and vertically, respectively, and overlaid with a measurement grid dividing the surface into four sections. For detailed guidance on image assessment, refer to the original publication by Luna et al. (2017). For the osteometric approach only the Ratios DE/AD, FI/CF and the Discriminant Function were measured. Segment lengths were extracted directly from the picture editor to calculate the DE/AD and FI/CF ratios, which along with the discriminant function informed sex classification (thresholds: 1.16 for DE/AD, 0.99 for FI/CF, and 0.078 for the discriminant function). Values below the threshold indicate for female sex, values above for male sex.

### 3.3 Osteometric Method (Schutkowski 1990)

**FIGURE 1:**
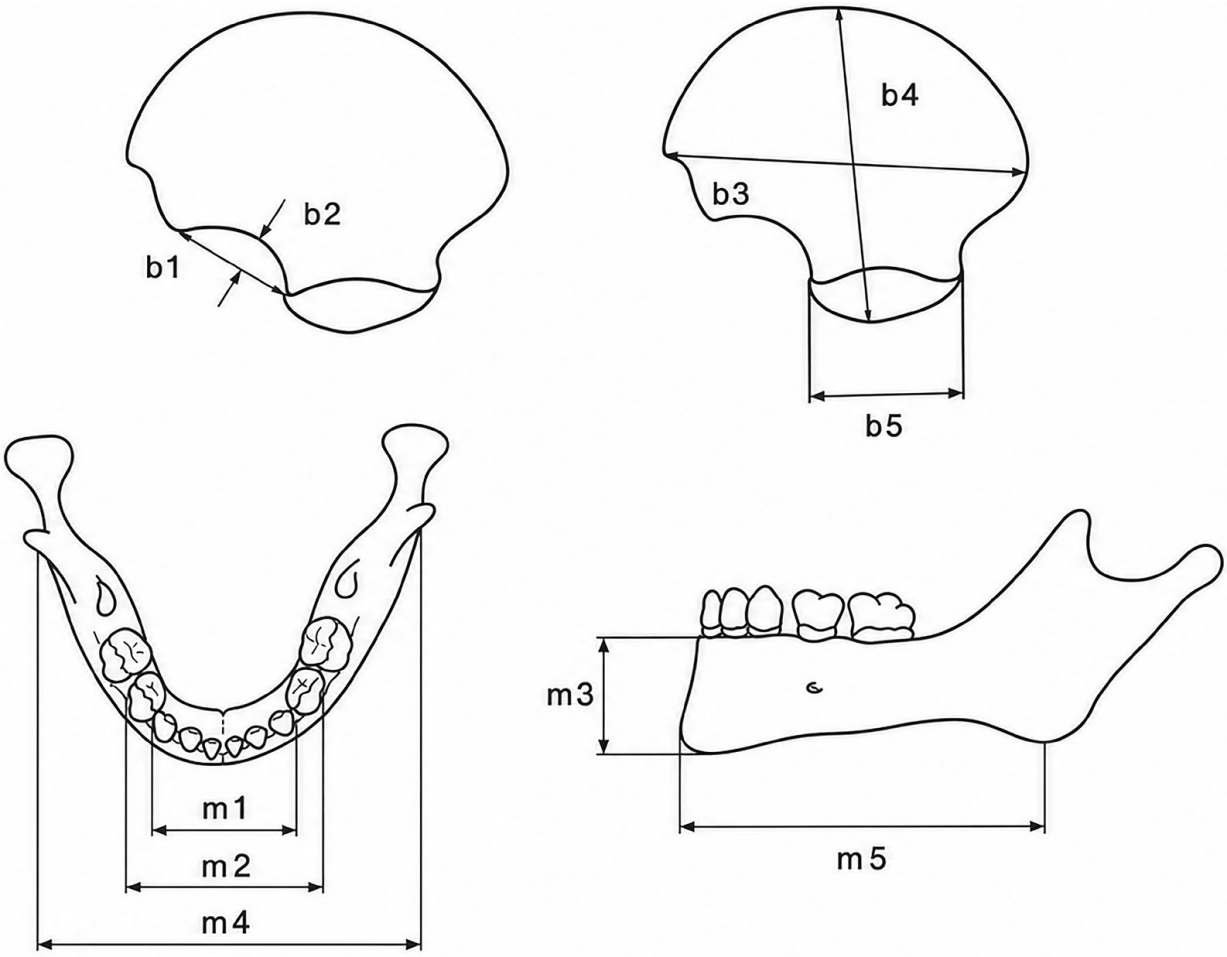
Measurement distances of the discriminant function analysis for sex determination on the Os ilium (top) and on the mandible (bottom). Adapted from: Schutkowski (1990).

Schutkowski's (1990) osteometric methods for sex estimation in subadults based on discriminant analysis of the mandible and the Os ilium. Different measurements (Figure 1) were taken on each skeletal element. Indices were calculated from these lengths and substituted into ten discriminant functions. All functions used a threshold of 0, with positive values classified as male and negative as female.

## 4 Results

When the age groups (0–24 months, infans I, and infans II) are examined separately (Table 2), the consistency of correct classification within a single method fluctuates, both within and across skeletal series, with significant variations observed across different skeletal series (up to 94.4%). Moreover, significant differences (up to 100%) exist between methods within an age group of a skeletal series. This is also reflected in the distribution of accurate sex allocation across skeletal series and methods presented in Table 3. There are notable differences in the accuracy of sex classification among methods and between sexes. None of the methodologies achieve a correct sex assignment rate deemed acceptable (> 75%) as per De Vito and Saunders (1990) and DiGangi and Moore (2012). When considering overall accuracy, rates range from 35.6% for Schutkowski's discriminant function 1 on the Mandibula to 65.0% for discriminant function 3 on the Os ilium by Schutkowski. If females and males are examined separately, correct sex classification varies widely, from 0.0% for females from Medenheim using Schutkowski's discriminant functions on the Os ilium to 100% for male individuals from the St. Nikolai skeletal series using Luna et al.'s discriminant function, and several of Schutkowski's discriminant functions on the Os ilium.

**TABLE 2:** Rate of correct classification by age at death in percent.

Skeletal series/age classes	Rate of correct classification																			
	Luna et al. (2017)						Discriminant functions by Schutkowski (1990)													
	Morphological		Osteometric				Os ilium											Mandibula		
	OM	*n*	Ratio AD/DF	Ratio CF/FI	DF	*n*	DF1	DF2	DF3	DF4	DF5	DF6	DF7	DF8	DF9	DF10	*n*	DF1	DF2	*n*
0–24 m																				
Gö C	—		—	—	—		—	—	—	—	—	—	—	—	—	—		—	—	
EL	77.8%	1	100%	33.3%	33.3%	1	0.0%	0.0%	100%	22.2%	88.9%	88.9%	66.7%	33.3%	22.2%	100%	1	55.6%	22.2%	1
MH	29.6%	5	55.6%	44.4%	66.7%	3	13.9%	13.9%	12.5%	6.95	4.2%	6.9%	12.5%	20.8%	13.9%	4.2%	5	63.0%	63.0%	4
*N*	—		—	—	—		—	—	—	—	—	—	—	—	—	—		—	—	
Inf I																				
Gö C	27.8%	2	66.7%	66.7%	83.3%	2	88.9%	88.9%	94.4%	88.9%	61.1%	88.9%	88.9%	94.4%	88.9	55.6%	2	33.3%	100%	1
EL	69.4%	3	0.0%	0.0%	0.0%	2	0.0%	0.0%	0.0%	38.9%	50.0%	38.9%	5.6%	0.0%	0.0%	72.2%	2	16.7%	33.3%	2
MH	29.6%	3	66.7%	66.7%	33.3%	1	25.0%	25.0%	50.0%	19.4%	8.3%	8.3%	16.7%	72.2%	25.0%	0.0%	3	18.5%	22.2%	3
*N*	33.3%	8	91.7%	66.7%	58.3%	8	51.9%	57.4%	67.7%	46.3%	49.1%	45.4%	59.3%	68.5%	51.9%	50.9%	8	55.6%	55.6%	2
Inf II																				
Gö C	—		—	—	—		—	—	—	—	—	—	—	—	—	—		—	—	
EL	—		100%	0.0%	50.0%	2	50%	50%	50%	55.6%	66.7%	56.6%	50%	0.0%	50%	61.1%	2	44.4%	50%	2
MH	—		66.7%	33.3%	66.7%	2	0.0%	0.0%	0.0%	0.0%	27.8%	0.0%	0.0%	16.7%	0.0%	50%	2	100%	44.4%	1
*N*	16.7%	2	66.7%	50.0%	16.7%	2	0.0%	0.0%	27.8%	0.0%	50.0%	38.9%	38.9%	38.9%	33.3%	88.9%	2	—	—	

Abbreviations: DF, discrimant function; EL, skeletal series of Eldagsen; Gö C, skeletal series of Göttingen Campus; MH, skeletal series of Medenheim; *n*, number of examined skeletal elements; *N*, skeletal series of St. Nikolai; OM, overall morphology.

**TABLE 3:** Rate of correct classification by skeletal series and sex for each method.

Method	Rate of correct classification								
Luna et al. (2017)	♂				♀				*t*
Facies auricularis left	Gö C	EL	MH	*N*	Gö C	EL	MH	*N*	
	(*n* = 1)	(*n* = 2)	(*n* = 2)	(*n* = 1)	(*n* = 0)	(*n* = 0)	(*n* = 3)	(*n* = 4)	(*n* = 13)
Morphological	33.3%	44.4%	22.2%	44.4%	—	—	29.6%	22.2%	31.0%
Osteometric									
*Ratio DE/AD*	66.7%	0.0%	100%	100%	—	—	33.3%	91.7%	64.6%
*Ratio FI/CF*	66.7%	50.0%	66.7%	66.7%	—	—	33.3%	50.0%	
*DF*	66.7%	66.7%	100%	100%	—	—	83.3%	33.3%	61.0%

Facies auricularis right	Gö C	EL	MH	*N*	Gö C	EL	MH	*N*	*t*
	(*n* = 1)	(*n* = 0)	(*n* = 1)	(*n* = 1)	(*n* = 0)	(*n* = 3)	(*n* = 3)	(*n* = 4)	(*n* = 13)
Morphological	22.2%	—	11.1%	22.2%	—	66.7%	29.6%	36.1%	31.3%
Osteometric									
*Ratio DE/AD*	66.7%	—	66.7%	66.7%	—	66.7%	33.3%	83.3%	63.9%
*Ratio FI/CF*	66.7%	—	66.7%	100%	—	11.1%	33.3%	66.7%	57.4%
*DF*	100%	—	100%	100%	—	11.1%	50.0%	50.0%	68.5%

Schutkowski (1990)	♂				♀				*t*
Os ilium left	Gö C	EL	MH	*N*	Gö C	EL	MH	*N*	
	(*n* = 1)	(*n* = 2)	(*n* = 2)	(*n* = 1)	(*n* = 0)	(*n* = 0)	(*n* = 4)	(*n* = 4)	(*n* = 14)
DF1	88.9%	50.0%	55.6%	88.9%	—	—	0.0%	44.4%	46.5%
DF2	88.9%	50.0%	55.6%	88.9%	—	—	0.0%	44.4%	46.5%
DF3	88.9%	50.0%	50.0%	88.9%	—	—	0.0%	52.8%	47.9%
DF4	88.9%	77.8%	27.8%	100%	—	—	0.0%	25.0%	43.1%
DF5	88.9%	77.8%	16.7%	100%	—	—	0.0%	25.0%	41.7%
DF6	88.9%	66.7%	27.8%	100%	—	—	0.0%	33.3%	43.8%
DF7	88.9%	50.0%	0.0%	88.9%	—	—	0.0%	50%	41.0%
DF8	100%	0.0%	50.0%	33.3%	—	—	16.7%	72.2%	45.1%
DF9	88.9%	50.0%	55.6%	88.9%	—	—	0.0%	38.9%	45.1%
DF10	33.3%	72.2%	16.7%	100%	—	—	13.9%	80.6%	51.4%

Os ilium right	Gö C	EL	MH	*N*	Gö C	EL	MH	*N*	*t*
	(*n* = 1)	(*n* = 0)	(*n* = 1)	(*n* = 1)	(*n* = 0)	(*n* = 3)	(*n* = 3)	(*n* = 4)	(*n* = 13)
DF1	88.9%	—	100.0%	100%	—	0.0%	0.0%	47.2%	56.0%
DF2	88.9%	—	100.0%	100%	—	0.0%	0.0%	47.2%	56.0%
DF3	88.9%	—	66.7%	100%	—	37.0%	0.0%	69.4%	60.3%
DF4	88.9%	—	77.8%	100%	—	18.5%	0.0%	25.0%	51.7%
DF5	88.9%	—	33.3%	88.9%	—	55.6%	0.0%	33.3%	50.0%
DF6	88.9%	—	33.3%	77.8%	—	48.1%	0.0%	44.4%	48.8%
DF7	88.9%	—	66.7%	100%	—	25.9%	0.0%	52.8%	55.7%
DF8	88.9%	—	88.9%	100%	—	11.1%	37.0%	63.8%	65.0%
DF9	88.9%	—	100%	100%	—	7.4%	0.0%	41.7%	56.3%
DF10	77.8%	—	0.0%	44.4%	—	74.1%	14.8%	88.9%	50.0%

Mandibula	Gö C	EL	MH	*N*	Gö C	EL	MH	*N*	*t*
	(*n* = 1)	(*n* = 2)	(*n* = 2)	(*n* = 1)	(*n* = 0)	(*n* = 3)	(*n* = 5)	(*n* = 1)	(*n* = 15)
DF1	33.3%	11.1%	5.6%	44.4%	—	7.4%	68.9%	6.7%	35.6%
DF2	100%	83.3%	33.3%	88.9%	—	33.3%	46.7%	22.2%	55.2%

Abbreviations: DF, discrimant function; EL, skeletal series of Eldagsen; Gö C, skeletal series of Göttingen Campus; MH, skeletal series of Medenheim; *n*, number of examined skeletal elements; *N*, skeletal series of St. Nikolai; OM, overall morphology.

### 4.1 Pelvis

Schutkowski's discriminant functions exhibit correct assignment rates ranging from 0.0% to 100%, displaying notable variability across different skeletal series, pelvis regions, and discriminant functions (Table 3). The correct sex assignment varies between the lowest value of 0.0% for discriminant functions 1–7 and 9 for female individuals of the Medenheim series and the highest value of 100% for discriminant functions DF4‐6 and DF10 of the St. Nikolai series, as well as DF8 of the University Campus skeleton series for the left Os ilium, and for discriminant functions DF1‐4 and DF7‐9 of the right Os ilium for male individuals. When considering only the correct overall reliability of the assignment, the values diverge from 41.0% (DF7, left Os ilium) to 65.0% (DF8, right Os ilium). Differences in correct classification between sexes are evident, showing a deviation of up to 80.6%. Female individuals generally exhibit a lower rate of correct classification in most functions and are more frequently misidentified as male. There is a tendency for larger deviations between the left and right Os ilium, with the right Os ilium generally exhibiting a slightly higher level of determination accuracy.

On the contrary, the morphological assessment of Luna and colleagues' evaluation of Facies auricularis demonstrates a contrasting trend. The left Facies auricularis displays higher rates of correct assessment. The overall morphology yields a total correct classification rate of 36.2%, with sex‐specific rates of 30.6% for males and 41.8% for females. The osteometric approach demonstrates an overall higher rate of correct classification ranging between 57.4% and 68.5%. When examined separately for males and females, the values within a skeletal series vary by up to 66% (Ratio DE/AD, Medenheim). For the skeletal series where a comparison between sexes is possible, it becomes apparent that male individuals are more frequently classified as male than female individuals are as female. However, assessments vary significantly among observers and within individual assessments by the same observer. Differences also emerge between the evaluation of the left and right Os ilium of the same individual by the same observer.

### 4.2 Mandible

The discriminant analysis method by Schutkowski applied to the mandible demonstrates a classification accuracy ranging from 35.6% (DF1) to 55.2% (DF2). Except for the female individuals of the Medenheim series, discriminant function 2 exhibits a higher rate of correct classification. Differences are evident both between sexes within a skeletal series (up to 63.3%) and across different skeletal series (up to 66.6%).

### 4.3 Intra‐ & Inter‐Observer

As detailed in Tables 4 and 5, the intra‐ and inter‐observer agreement tests demonstrate that the intra‐observer agreement is more substantial than the inter‐observer agreement. However, all methods indicate that there is limited agreement among and between the observers. While in a few instances a coefficient above 0.8 was achieved, indicating almost perfect agreement, the majority of comparisons resulted in fair to moderate agreement, with some showing only slight agreement (< 0.2). The inter‐observer error did not consistently favor one methodology over another, with some tests displaying higher agreement for Luna and colleagues' osteometric approach and others favoring Schutkowski's discriminant functions. However, the intra‐observer error clearly favored an osteometric approach over a morphological approach.

**TABLE 4:** Inter‐observer error results for each method assessed by k coefficient.

Method				R1vs.R2	R1vs.R3	R2vs.R3
Luna et al. (2017)	Osteometry	Facies auricularis Left	*Ratio AD/DF*	0.82	0.421	0.312
			*Ratio CF/FI*	0.542	0.247	−0.0548
			*DF*	−0.0377	0.621	0.0678
		Facies Auricularis Right	*Ratio AD/DF*	0.676	−0.0909	0.385
			*Ratio CF/FI*	−0.161	−0.171	0.351
			*DF*	0.122	−0.154	0.526

				R1vs.R2	R1vs.R3	R2vs.R3
Schutkowski (1990)	Discriminant analysis	Os ilium left	*DF 1*	0.545	0.462	0.526
			*DF 2*	0.561	0.493	0.545
			*DF 3*	0.587	0.552	0.471
			*DF 4*	0.156	0.357	−0.125
			*DF 5*	0.198	0.292	−0.0678
			*DF 6*	0.6	0.5	−0.1667
			*DF 7*	−0.138	0.545	0.22
			*DF 8*	0.746	0.83	0.739
			*DF 9*	0.664	0.614	0.44
			*DF 10*	0.108	0.588	0.364
		Os ilium right	*DF 1*	0.532	0.459	0.602
			*DF 2*	0.532	0.459	0.602
			*DF 3*	0.6	0.533	0.638
			*DF 4*	0.356	0.4	0.169
			*DF 5*	0.167	0.455	0.167
			*DF 6*	0.312	0.455	0.211
			*DF 7*	0.444	0.78	0.5
			*DF 8*	0.727	0.526	0.769
			*DF 9*	0.453	0.814	0.453
			*DF 10*	0.094	0.169	0.00613

				R1vs.R2	R1vs.R3	R2vs.R3
		Mandibula	*DF 1*	0.437	−0.101	−0.0315
			*DF 2*	0.604	0.696	0.483

Abbreviations: DF, discriminant function; R1–3, observer 1–3; vs., versus.

**TABLE 5:** Intra‐observer error results for each method assessed by k coefficient.

Method				R1	R2	R3
Luna et al. (2017)	Morphology	Facies auricularis left	*OM*	0.053	0.091	−0.17
		Facies auricularis right	*OM*	0.17	0.032	−0.12

				R1	R2	R3
Schutkowski (1990)	Discriminant analysis	Os ilium left	*DF 1*	0.526	0.583	0.722
			*DF 2*	0.526	0.583	0.722
			*DF 3*	0.426	0.583	0.8525
			*DF 4*	1.0	−0.08	0.571
			*DF 5*	0.809	−0.08	0.571
			*DF 6*	1.0	1.0	0.704
			*DF 7*	0.471	0.757	0.644
			*DF 8*	0.664	0.486	0.655
			*DF 9*	0.438	0.71	0.809
			*DF 10*	0.75	0.788	1.0
		Os ilium right	*DF 1*	1.0	0.17	0.684
			*DF 2*	1.0	0.17	0.684
			*DF 3*	1.0	0.711	0.609
			*DF 4*	0.571	0.393	0.529
			*DF 5*	0.876	0.614	0.286
			*DF 6*	0.733	0.393	0.577
			*DF 7*	0.8571	0.412	0.586
			*DF 8*	1.0	0.738	0.759
			*DF 9*	0.819	0.723	0.824
			*DF 10*	0.44	0.478	0.587

				R1	R2	R3
		Mandibula	*DF 1*	0.608	−0.0476	0.842
			*DF 2*	0.88	0.759	0.727

Abbreviations: DF, discriminant function; OM, overall morphology; R1–3, observer 1–3.

## 5 Discussion

In recent decades, methodologies for differentiating between skeletons of male and female subadults based on skeletal characteristics have been continuously developed. These are predicated on the assumption that sexual dimorphism manifests during the prenatal phase and is subsequently established in early childhood due to the differential production and secretion of sex hormones (Lanciotti et al. 2018; H. Cardoso 2005; Knickmeyer and Baron‐Cohen 2006; Stull et al. 2017; Loth and Henneberg 2001). Given the frequent utilization of the pelvis and cranium in the sex estimation of adult skeletal remains, studies were conducted into the potential of skeletal features of the pelvis and cranium in the sexing of subadults (Sutter 2003; Schutkowski 1990; Loth and Henneberg 2001). Although these studies indicate the potential of skeletal features in sex estimation, the reliability of such methods remains a point of contention. The validity of numerous sex estimation techniques varies significantly between original research and subsequent validation efforts. For instance, methods reliant on morphometric data frequently demonstrate superior accuracy in the original studies, whereas their reproducibility often declines in independent validation studies (Monge Calleja et al. 2023). The results of this study align with those previously reported by Monge Calleja and colleagues, thereby reinforcing the imperative to conduct a comprehensive evaluation of the applicability, replicability and transferability of the methodologies employed for sex estimation in subadults based on skeletal characteristics. While these methods offer significant potential, they also present challenges that necessitate careful consideration, particularly when applied across diverse populations. The current study emphasizes the significance of dependable and precise techniques in forensic anthropology and bioarchaeology, particularly in the evaluation of subadult remains, where sexual dimorphism is often less discernible due to the development of sexually dimorphic features during adolescence (Scheuer and Black 2000).

Despite their limitations, morphological and morphometric approaches remain practical diagnostic tools, especially in contexts where time and resources for DNA analysis are constrained. These methods, which rely on the visual assessment of skeletal features or precise measurements, provide a viable alternative in forensic and archaeological scenarios where immediate results are necessary (Grosskopf 2004; Seyed Ali Agha 2009). For instance, in cases where DNA is poorly preserved due to degradation or where destructive sampling of valuable archaeological specimens is to be avoided, morphological methods offer a non‐invasive solution. Additionally, their cost‐effectiveness and efficiency make them particularly advantageous in resource‐limited settings or when rapid preliminary assessments are required, such as in forensic investigations of mass graves (VanBaarle 2019). However, the inherent subjectivity of morphological assessments poses a significant challenge, as different observers may interpret skeletal characteristics differently, leading to variability in results. In contrast, osteometric measurements, being more objective, tend to yield more consistent results, particularly in intra‐observer comparisons. Recent studies have demonstrated that advancements in imaging technologies, such as 3D modeling and geometric morphometrics, offer promising alternatives to traditional methods. These approaches can enhance the accuracy of sex estimation by capturing subtle differences in skeletal morphology that may not be discernible through conventional visual assessments (Monge Calleja et al. 2023). However, their practical application is often limited by high costs, the requirement for specialized equipment, and the need for extensive technical expertise.

Before examining the specific factors underlying the observed variability in method accuracy, it should be noted that the sample size of the present study represents an inherent constraint on the interpretation. The percentages reported are in several cases based on small sample sizes. Therefore, the results should be read as indicative patterns rather than definitive benchmarks. The consistent directionality of findings across independent skeletal series does, however, support the validity of the study's core conclusions. With this in mind, the following sections examine the demographic, population‐specific, and methodological factors that may account for the observed variations.

### 5.1 Demographic and Age‐Related Considerations

The initial samples of Luna's (Luna et al. 2017, 2021) and Schutkowski's (1990) studies exhibited a male bias, which reflects a trend observed in certain populations as the mortality rate is higher in male infants than in females due to biological vulnerabilities and environmental risks (Pongou 2013). However, an elevated mortality rate among male infants and the resulting overrepresentation in samples may introduce potential distortions in the findings. The development of morphological methodology is particularly susceptible to this issue. As the proportion of male individuals in a sample increases and is greater than the number of females, the likelihood of observing a characteristic of males also increases. Consequently, if the number of females in the sample is less than that of males, the probability of correctly identifying a masculine trait is greater than it would be in a real population (Olivares and Aguilera 2015). This bias highlights the necessity of considering demographic factors, such as sex distribution, during the development of these methods to avoid sex‐specific distortions. In addition to the distribution of sex, a variety of other demographic characteristics, including the age at death of an individual, exert a decisive influence on the rate of correct classification.

Whilst no distinct age trends were identified in the present study, insights into the impact of age‐related skeletal development on the precision of sex estimation methods can be derived from reference and validation studies. For instance, Luna et al. (2021) documented relatively high accuracies in the prenatal period and in early childhood (0–2 years), particularly for specific metric traits such as FI/CF and DE/AD ratios, which attained accuracies of 80%–90%. Morphological characteristics such as Overall Morphology (OM) were slightly less effective but still achieved good results (70%–86%). A slight decrease in accuracy was observed in the age group 2.1–5 years, which the authors attributed to a lower expression of sexual dimorphism during further skeletal growth. The findings reveal a divergence from the trends previously documented by Marino et al. (2021) for older age groups (4–17 years), wherein morphological traits exhibited a consistent superiority over metric traits. In the younger age group of 4–12 years, morphological methods attained the highest accuracies (77%–86%), a phenomenon attributed by the authors to the stabilization of dimorphic traits and the emergence of hormonal influences. In adolescents (13–17 years), morphological traits demonstrated a reliability of approximately 78%, while the accuracy of metric traits exhibited a slight increase but remained variable. The increase in the reliability of identification with the age at death of the individuals was also demonstrated in studies by the working group at the Johann‐Friedrich‐Blumenbach‐Institute of Zoology and Anthropology, Department of Historical Anthropology and Human Ecology at the University of Göttingen (Ahlbrecht et al. 2025).

The interaction between age, skeletal development and method effectiveness underscores the challenges inherent in implementing a uniform approach across diverse age groups. On the one hand, certain methods yield excellent results in early childhood; however, their efficacy may diminish as skeletal maturation and stabilization of dimorphism progresses, as evidenced by the disparities observed between skeletal series. Consequently, a diversified approach that incorporates age‐dependent trends and the characteristics of the respective skeletal series is imperative to enhance the precision of sex estimation across diverse developmental stages.

### 5.2 Population‐Specific Considerations in Sex Estimation

Another critical aspect highlighted by the study is the importance of considering population‐specific characteristics when developing sex estimation methods. The accuracy of the classification varies considerably between the populations examined in the study as well as in comparison to the reference skeletal series. This discrepancy could be attributed to the presence of varying degrees of sexual dimorphism in subadults in the regions of origin. This finding aligns with other research indicating that sexual dimorphism varies significantly across populations. For example, studies comparing French, Portuguese, and English populations have documented interpopulation differences in pelvic and auricular surface features, further complicating sex estimation across diverse populations (Walker 2005; Rmoutilová et al. 2017). This assertion may also be applicable to the mandible and viscerocranium. Although it can be stated that the dimensions develop almost completely up to the age of five years and then correspond nearly to the adult dimensions (Scheuer and Black 2004), numerous studies have shown morphological variation between different geographical groups of origin (Relethford 1994; Relethford and Harding 2001; Hennessy and Stringer 2002). Analogous to the variating dimorphism of pelvic traits between populations, this variation in trait expression may have led to reduced correct determinations. In addition to the different geographical origins, two of the skeletal series analyzed in the study originate from a different time period. The methodologies employed in the present study for determining sex were developed using samples from the 19th and 20th centuries in Portugal (Luna et al. 2017) and from the 18th and 19th centuries in England (Schutkowski 1990). However, the Medenheim and St Nikolai skeletal series originate from the medieval period. These temporal differences underscore the impact of secular trends on skeletal morphology, such as changes in nutrition, lifestyle, and health over time, which may influence the expression of sexual dimorphism. Research has shown that secular trends can affect key traits like bone robusticity and pelvic dimensions, suggesting the need for chronological specificity when applying sex estimation methods (H. F. Cardoso 2008; Viciano et al. 2020).

### 5.3 Variability in Method Transferability and Reproducibility

The present study also raises serious concerns about the transferability and reproducibility of the evaluated sex estimation methods. Critically, the Cohen's kappa values reveal that, in the majority of comparisons, inter‐observer agreement reached only slight to fair levels, with moderate agreement achieved only in isolated cases. This constitutes a substantial finding. The methods evaluated are, to a large extent, strongly observer‐dependent and results obtained by one observer cannot in many cases be directly compared to those of another. This degree of observer variability limits the reliability of these methods in practice. It questions the methods' applicability in contexts where reproducibility is essential, such as forensic casework or bioarchaeological analysis.

For instance, while Luna et al. (2017) methodology, characterized by low inter‐observer error, showed promising results, the current study found varying levels of agreement between observers, ranging from fair to moderate. This variability may be attributed to the subjective nature of morphological assessment which is susceptible to individual differences in interpretation. Preservation state of the remains constitutes an additional confounding factor, as skeletal features can be affected by post‐mortem modifications and morphological degradation (White and Folkens 2005). Therefore, poorly preserved remains can lead to inaccurate evaluations, complicating the determination process further. Notably, the intra‐observer error was consistently lower than inter‐observer error across all methods, suggesting that the methods can be applied with greater internal consistency by a single observer. This distinction has direct implications for study design, particularly where multiple observers are involved.

The current study also examined the preference for using one side of the pelvis over the other in sex estimation but found no consistent superiority of one side. Additionally, it was determined that the left and right sides of an individual's body exhibited opposing sex‐specific characteristics. If, as in the reference studies (Luna et al. 2017, Schutkowski 1990), only the left side of the pelvis had been considered, this would have resulted in erroneous sex estimation in specific instances. Therefore, the study's findings indicate that both sides are viable, particularly when considering the frequent erosion of the Os ilium in archaeological contexts.

Taken together, these results highlight the urgent need for systematic inter‐observer reliability testing in future method development and validation studies, as well as the establishment of standardized training protocols to reduce variability in morphological assessments. Machine learning and statistical modeling approaches have been proposed as tools to enhance reproducibility by minimizing human biases in trait evaluation (Nikita and Nikitas 2020).

## 6 Conclusion

In summary, the study highlights the complexity and challenges associated with sex estimation based on skeletal features, particularly in subadult remains. While promising methods exist, the observed differences between populations underscore the ongoing challenge of developing universally applicable methods for sex estimation. These differences raise important questions about the extent to which existing methods need to be refined and adapted and highlight the urgency of new methods and the need for further research in this area. Future studies should focus on developing more reliable and accurate methods that take into account demographic, developmental, and socio‐cultural factors. In addition, the integration of more objective, quantitative methods together with continued refinement of morphological assessments could improve the accuracy and applicability of these techniques in forensic and archaeological practice. The potential of hybrid approaches that combine morphological, osteometric, and imaging data should also be explored, as they may offer a more holistic framework for addressing the limitations of current methods.

## Author Contributions

**Clara L. Behrendt:** writing – original draft, writing – review and editing, data curation, investigation, formal analysis. **Birgit Grosskopf:** writing – review and editing, conceptualization, project administration, supervision. **Janine Mazanec:** writing – review and editing, supervision, project administration. **Katharina M. J. Tenorio:** writing – original draft, writing – review and editing, data curation, investigation. **Susanne Hummel:** writing – review and editing, conceptualization, supervision, project administration. **Susanna Hixt:** data curation, investigation, writing – review and editing.

## Funding

This work was supported by the Open Access Publication Fund of the University of Göttingen.

## Ethics Statement

This research involves historical human remains curated at the Historical Anthropology Collection at the University of Göttingen (Germany), a permanent collection dedicated primarily to teaching and research. The skeletal material originates from archaeological excavations carried out at the end of the 20th and early 21st centuries in Germany. These remains were not designated for reburial and were therefore transferred to the collection for scientific purposes in accordance with applicable German law and institutional regulations. Formal written permission to access and study the remains was granted by the collection's curatorial authority at the University of Göttingen. Research and handling of the remains were conducted in strict accordance with the ethical principles governing the handling of human remains and local institutional guidelines. Because the remains derive from historical archaeological contexts, no specific currently living descendant could be identified. Consequently, active community consultation was not feasible. However, this circumstance does not diminish the ethical obligation to treat the deceased individuals with respect and dignity throughout all stages of sample collection, analysis, and publication. Dissemination of the research results will be managed through peer‐reviewed publication and the university's public collection registry to ensure communication with the broader scientific community and the general public.

## Consent

The authors have nothing to report.

## Conflicts of Interest

The authors declare no conflicts of interest.

## Data Availability

The data that support the findings of this study are available in the main text of this article. The raw data supporting the findings of this study have been deposited in the Zenodo repository (DOI: https://doi.org/10.5281/zenodo.20677183). Access is restricted to protect the privacy of and integrity of sensitive skeletal data associated with historical skeletal remains. Data will be made available to qualified researchers upon request to the corresponding author, subject to institutional guidelines of the Historical Anthropology Collection at the University of Göttingen.
